# Safety and efficacy of narsoplimab in pediatric and adult patients with transplant-associated thrombotic microangiopathy: a real-world experience

**DOI:** 10.1038/s41409-024-02305-3

**Published:** 2024-05-21

**Authors:** Marta Castelli, Maria Caterina Micò, Anna Grassi, Alessandra Algarotti, Federico Lussana, Maria Chiara Finazzi, Benedetta Rambaldi, Chiara Pavoni, Giuliana Rizzuto, Paola Tebaldi, Francesca Vendemini, Marta Verna, Sonia Bonanomi, Andrea Biondi, Adriana Balduzzi, Alessandro Rambaldi, Giacomo Gotti

**Affiliations:** 1 Department of Oncology and Hematology, University of Milan and Azienda Socio-Sanitaria Territoriale Papa Giovanni XXIII, Bergamo, Italy; 2 Department of Oncology and Hematology, Azienda Socio-Sanitaria Territoriale Papa Giovanni XXIII, Bergamo, Italy; 3 Department of Pathology, Azienda Socio-Sanitaria Territoriale Papa Giovanni XXIII, Bergamo, Italy; 4 Department of Pediatrics, Fondazione IRCCS San Gerardo, Monza, Italy; 5 Department of Medicine and Surgery, University of Milano-Bicocca, Monza, Italy

**Keywords:** Immunotherapy, Cancer stem cells

## Abstract

Transplant-associated thrombotic microangiopathy (TA-TMA) is a severe complication following hematopoietic stem cell transplantation (HSCT). No approved treatments are currently available. This study presents real-world data obtained with narsoplimab, a human immunoglobulin G4 monoclonal antibody that inhibits MASP-2, the effector enzyme of the lectin pathway of the complement system. Between January 2018 and August 2023, 20 (13 adult and 7 pediatric) patients diagnosed with TA-TMA received narsoplimab under an ongoing compassionate use program. The diagnosis was based on internationally defined criteria for pediatric and adult patients. Fifteen patients fulfilled the criteria recently established by an international consensus on TA-TMA. Nineteen patients exhibited high-risk characteristics. Thirteen patients (65%) responded to narsoplimab, achieving transfusion independence and significant clinical improvement. The one-hundred-day Overall Survival (OS) post-TA-TMA diagnosis was 70%, and 100% for responders. Narsoplimab proved to be effective and safe in the treatment of high-risk TA-TMA, with no increased infectious complications or other safety signals of concern across all age groups. The high response rates and the encouraging survival outcomes underscore the potential of narsoplimab as a valuable therapeutic option, particularly for high-risk cases.

## Introduction

Transplant-associated thrombotic microangiopathy (TA-TMA) occurs in up to one-third of pediatric and adult patients following hematopoietic stem cell transplantation (HSCT) [1]. TA-TMA is characterized by endothelial damage and complement activation, which determine microangiopathic hemolytic anemia, thrombocytopenia, and thrombosis, leading to organ dysfunction, high morbidity, and mortality [2, 3].

Several diagnostic criteria have been proposed including the presence of anemia, thrombocytopenia, elevated lactate dehydrogenase (LDH), and schistocytes [1, 4–6]. The lack of harmonization in the diagnostic criteria has precluded multi-institutional studies aimed at better understanding the incidence of TA-TMA. To address this issue, an expert panel recently reviewed available criteria to facilitate the early identification of TA-TMA and reached a consensus, updating both diagnostic and prognostic criteria [7].

Treatment of TA-TMA remains an unmet clinical need. There is common agreement on avoiding toxic serum concentration of calcineurin inhibitors (CNIs) [8] and tapering their dosage as soon as the laboratory evidence of endothelial damage becomes available. Much less agreement on CNI withdrawal exists when a formal diagnosis of TA-TMA is posed because of the risk of inducing or exacerbating acute GvHD, a known trigger of endothelial damage and TA-TMA [9–11].

Various therapeutic strategies have been explored for TA-TMA, however, their efficacy has been limited [12–14]. Given the current understanding of complement activation as a fundamental driver of TA-TMA [15–17], it is not surprising that many new investigational therapies target the complement system [18, 19], even if an increased risk of infectious complications may hamper the clinical benefit of C5 inhibitors [20]. In addition, none of these therapies are currently approved for TA-TMA.

Narsoplimab (Omeros Corporation, Seattle, USA) is a fully human immunoglobulin G4 monoclonal antibody that inhibits mannan-binding lectin-associated serine protease-2 (MASP-2), the effector enzyme of the lectin pathway of the complement system [21, 22]. MASP-2 inhibition leaves intact the lytic arm of the classical pathway of complement. In allogeneic transplant recipients, targeting the lectin pathway without affecting the lytic function of the classical pathway may potentially preserve the adaptive immune defense mechanisms, providing protection against encapsulated organisms, like *Neisseria meningitidis* [23]. In a recent single-arm open-label pivotal trial, patients with TA-TMA received intravenous narsoplimab. The drug proved safe, significantly improved laboratory TMA markers, and resulted in clinical response and favorable overall survival [24].

Here we describe a retrospective series of pediatric and adult patients with severe TA-TMA treated with narsoplimab under a compassionate use program made available based on the results of the recently published pivotal trial.

## Material and methods

### Patients

Pediatric and adult patients with TA-TMA were treated with narsoplimab under a compassionate use, following approval from the Ethical Committee. Informed consent was obtained from all subjects. Pediatric patients were transplanted at the Pediatric HSCT Unit of Fondazione IRCCS San Gerardo dei Tintori in Monza, Italy, while adults at the Bone Marrow and Transplant Unit of Papa Giovanni XXIII Hospital in Bergamo, Italy.

Before the publication of the expert panel’s consensus [7], pediatric patients were diagnosed with TA-TMA using the criteria established by Jodele et al. [1]. For adult patients, the diagnosis was based on the criteria proposed by Cho et al. [6]. Starting in 2023, the updated criteria by Schoettler et al. [7] were applied for newly diagnosed TA-TMA cases in both pediatric and adult patients. Furthermore, patients previously diagnosed with TA-TMA using the criteria from Jodele and Cho were reevaluated according to the recent revised criteria.

In all patients the diagnosis of TA-TMA was supported by the presence of at least 4 of the following criteria: (1) anemia, defined as failure to achieve transfusion independence despite neutrophil engraftment; hemoglobin decline from patient’s baseline by 1 g/dL or new-onset transfusion dependence; (2) thrombocytopenia, defined as failure to achieve platelet engraftment, higher-than-expected transfusion needs, refractory to platelet transfusions, or 50% reduction in baseline platelets count after full platelet engraftment; (3) LDH exceeding the upper limit of normal (ULN); (4) presence of schistocytes; (5) hypertension, (6) reduced haptoglobin level, and (7) proteinuria, defined as a random urine protein-to-creatinine ratio (rUPCR) of 1 mg/mg [7]. In accordance with the diagnostic criteria outlined by Cho et al., a positive Coombs Test was considered an exclusion criterion for TA-TMA diagnosis.

### High-risk TA-TMA

According to the most recent consensus, patients were retrospectively classified as high-risk based on the presence of at least one of the following poor prognostic features: (1) rUPCR ≥ 1 mg/mg; (2) the presence of organ dysfunction; (3) LDH levels exceeding two times the ULN; (4) concurrent grade II–IV acute GvHD; (5) concurrent infections (bacterial or viral) [7]. In the absence of these features, TA-TMA was considered standard-risk.

### Treatment schedule

Narsoplimab was planned for intravenous infusion at a dose of 4 mg/kg twice weekly in both adult and pediatric patients for at least 8 doses, on the basis of evidence that this dosage provided well greater than 80% inhibition of the lectin pathway [24]. In case of incomplete response and in the absence of any toxicity, treatment could be prolonged with no limit to the number of infusions. For one patient, the dosage was increased to 3 times a week due to a poor initial response to the treatment.

Clinical and laboratory parameters, including blood counts, haptoglobin, schistocytes, total and fractionated bilirubin, transaminases, creatinine, LDH, C-reactive protein (CRP) levels, and spot proteinuria (protein/creatinine ratio) were collected from all narsoplimab-treated patients per standard clinical practice. Routine blood examinations were collected prior to each narsoplimab dose and then twice weekly.

### Response criteria

Response criteria to narsoplimab therapy were defined by improvement in both laboratory TMA markers and any one of several potential clinical benefits at any time after the first dose of narsoplimab, as described by Khaled et al. [24]. Time to hematologic response was defined as the time from first narsoplimab administration to the first achievement of both platelet count and LDH response criteria.

### Statistical analysis

Only patients with TA-TMA were included in the study. Data were presented as number with percentage for categorical variables and as mean or median with range for continuous ones. Overall survival (OS) and one-hundred-day survival were calculated from TA-TMA diagnosis or from transplant date to death for any cause or last follow-up. OS and 100-day survival were estimated using the Kaplan–Meier method. Statistics were performed with R software, version 4.3.0.

## Results

### Patients’ characteristics and diagnosis

Between January 2018 and August 2023, 20 patients received narsoplimab for TA-TMA at the dose of 4 mg/kg. Thirteen patients were adults, with a median age of 40 (range, 32–71), and 7 patients were pediatric, with a median age of 13 (range, 5–19). All patients except two pediatric patients with sickle cell disease underwent HSCT for hematologic malignancies. Demographic and transplant characteristics are described in Table 1.

**Table 1:** Demographic and transplant patient characteristics.

Variable	Overall (N = 20)	Adults (N = 13)	Pediatrics (N = 7)
Age (years), median (range)	26.5 (5–71)	40 (32–71)	13 (5–19)
Male, No. (%)	8 (49)	5 (38)	3 (43)
Time from HSCT to TA-TMA diagnosis, days (range)	85.5 (24–452)	89 (30–452)	78 (24–246)
Time from current TA-TMA diagnosis to day 1, days (range)	3.5 (0–52)	3 (0–14)	7 (0–52)
Cell source, No. (%)			
Bone marrow	5 (25)	0 (0)	5 (71)
Peripheral blood	14 (70)	12 (92)	2 (29)
Cord blood	1 (5)	1 (8)	0 (0)
Matched donor, No. (%)			
Matched related	5 (25)	0 (0)	5 (71)
Matched unrelated	5 (25)	5 (38)	0 (0)
Mismatched donor, No. (%)			
Mismatched related	3 (15)	1 (8)	2 (29)
Mismatched unrelated	7 (35)	7 (54)	0 (0)
ABO incompatibility, No. (%)			
Major	6 (30)	4 (31)	2 (29)
Minor	3 (15)	3 (23)	0 (0)
Bidirectional	1 (5)	1 (8)	0 (0)
None	10 (50)	5 (38)	5 (71)
Conditioning regimen, No. (%)			
MAC	11 (55)	4 (31)	7 (100)
RIC	9 (45)	9 (69)	0 (0)
GvHD prophylaxis regimen, No. (%)			
ATG, CSA, MTX	11 (55)	9 (69)	2 (29)
CSA, MMF, PTCy	4 (20)	2 (15)	2 (29)
ATG, FK, MTX	1 (5)	1 (8)	0
ATG, MMF, CSA	1 (5)	1 (8)	0
CSA, MMF, MTX	1 (5)	0	1 (14)
CSA, MMF	1 (5)	0	1 (14)
CSA, MTX	1 (5)	0	1 (14)
Disease status at the time of transplant, No. (%)			
Active disease	9 (45)	7 (54)	2 (29)
CR1	6 (30)	4 (31)	2 (29)
CR2 or more	5 (25)	2 (15)	3 (43)
Baseline^a^ platelet count, ×10^9^/L			
≤20, No. (%)	13 (65)	7 (54)	6 (86)
>20, No. (%)	7 (35)	6 (46)	1 (14)
Baseline hemoglobin, g/dL (range)	9.1 (7.7–12.3)	9.1 (7.8–11.2)	9.4 (7.7–12.3)
Baseline platelets, per microliter (range)	16,000 (13,000–62,000)	18,000 (10,000–41,000)	14,000 (3000–62,000)
Baseline LDH, U/L (range)	531 (237–1201)	495 (237–1201)	702 (460–1073)
Acute GvHD ≥ overall grade 2, No. (%)	17 (85)	11 (86)	6 (86)
Infection, No. (%)	15 (75)	8 (61)	7 (100)

Values are presented as the number of patients with the corresponding percentage relative to the total (20 patients), adult (13 patients), and pediatric (7 patients) populations.

*LDH* lactate dehydrogenase, *MAC* myeloablative conditioning regimen *RIC* reduced intensity conditioning regimen, *CR* complete remission, *GvHD* graft versus host disease, *ATG* anti-thymocyte globulin, *CSA* cyclosporine, *MMF* mycophenolate mofetil, *FK* tacrolimus, *PTCy* post-transplant cyclophosphamide, *MTX* Methotrexate.

^a^Baseline: at time of TA-TMA diagnosis.

All the pediatric and adult TA-TMA were diagnosed according to the criteria proposed by Jodele and Cho, respectively. In addition, 15 (75%) patients fulfilled the criteria recently proposed by the Schoettler et al. expert panel group. The frequencies of the different diagnostic criteria met in our study population are reported in Table 2.

**Table 2:**
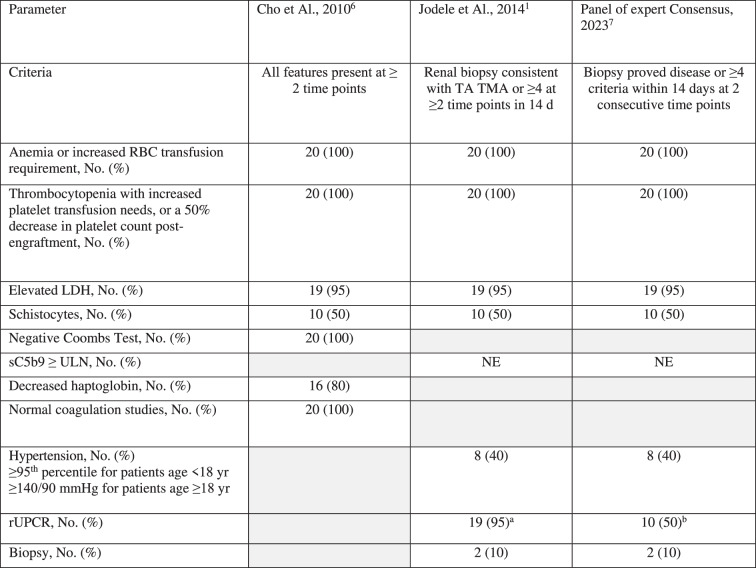
Incidence of TA-TMA according to multiple diagnostic criteria.

Values are presented as the number of patients meeting the specific diagnostic criterion with the corresponding percentage within the total population (20 patients). Gray boxes indicate that the criterion does not apply.

*RBC* red blood cells, *LDH* lactate dehydrogenase, *ULN* upper limit of normal, *sC5b-9* soluble terminal complement (membrane attack) complex (C5b-9), *rUPCR* random urine protein-to-creatinine ratio, *NE* not evaluated.

^a^rUPCR positive (> 2 mg/mg).

^b^rUPCR ≥ 1 mg/mg.

**Fig. 1:**
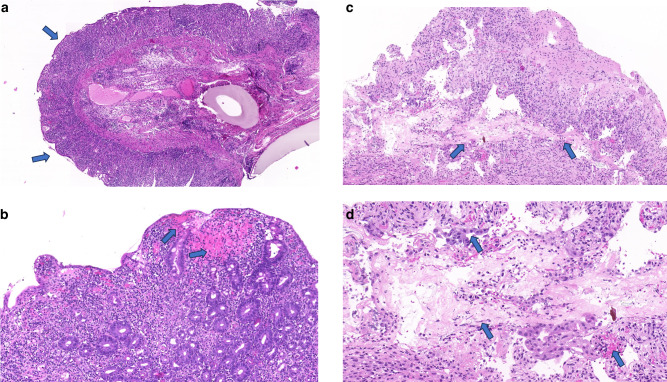
Histological confirmation of TA-TMA on intestinal (sigmoid A/B) and gastric (C/D) biopsy in two adult patients. **a** Loss of glands in the colon mucosa, characterized by the disappearance and atrophy of glands. Mucosal denudation, involving the loss of surface epithelium, often replaced by a single layer of regenerative enterocytes. **b** Widespread sloughing of epithelial cells and perivascular hemorrhages in the mucosa, indicated by the extravasation of red blood cells (RBC) from capillaries in the lamina propria. **c** Necrosis of the gastric mucosa and extensive ulceration. **d** Endothelial cell swelling, characterized by the enlargement of endothelial cells in the capillaries of the lamina propria. Sparse fungal elements in the vast necrotic area. Diffuse perivascular mucosal hemorrhage.

In our study, TA-TMA was confirmed by histologic biopsies in two adult patients. In the first case, the patient received the diagnosis of TA-TMA due to the presence of anemia, thrombocytopenia, elevated LDH, low haptoglobin, and proteinuria. Due to an intestinal perforation, this patient underwent an urgent colon resection. The biopsy of the resected sigmoid tract revealed typical signs of thrombotic microangiopathy, including gland loss, mucosal hemorrhages, and endothelial cell denudation, as commonly described in the literature [25] (Fig. 1a/b). After the surgical intervention, the patient started treatment with narsoplimab. Laboratory evidence of progressive response to treatment was documented and, following 11 doses, the patient achieved a complete laboratory and clinical response and is currently alive in complete remission.

In the second case, the diagnosis of TA-TMA was posed due to the onset of anemia, thrombocytopenia, elevated LDH, uncontrolled hypertension, and neurological symptoms such as confusion, tremors, and lethargy. The clinical picture was further complicated by an invasive fungal infection. As the patient began to experience melena, an esophagogastroduodenoscopy was performed. The patient was under treatment with CNI (tacrolimus) and steroids (methylprednisolone 1 mg/kg). The histologic examination revealed a deepithelialized mucosa with erosions, making it difficult to exclude either gastrointestinal acute GvHD (aGvHD) or ischemic damage from intestinal TA-TMA (iTAM) [26]. Fungal hyphae and necrotic areas were also identified in the biopsy, indicating potential outcomes of widespread endothelial damage (Fig. 1c/d). Despite iTAM presenting with clinical symptoms resembling those of intestinal GvHD and often coexisting with intestinal aGvHD—a notable trigger for TA-TMA [9, 27–29]—our patient exhibited persistent gastric bleeding unresponsive to immunosuppressive therapy. This patient did not respond to narsoplimab and died 78 days after the diagnosis of TA-TMA.

Median time from transplant to TA-TMA was 85.5 days (range, 24–452). Risk factors for TA-TMA development such as GvHD and infections were present in a high proportion of patients. Before the diagnosis of TA-TMA and treatment with narsoplimab, 19 of the 20 patients (95%) were diagnosed with high-risk TA-TMA according to the consensus criteria in Schoettler et al. [7]. Specifically, 17 patients (85%) had a diagnosis of grade II–IV acute GvHD at a mean of 52 days (range, 14–223 days) after transplant. Sixteen patients (80%) required the start of high-dose steroids as first-line treatment and a complete GvHD response was obtained in 6 of them (5 adult patients and 1 pediatric). The remaining 10 (50%) patients were deemed steroid-refractory and started a second-line therapy for acute GvHD, ruxolitinib in most cases.

Before narsoplimab, infections were documented in 15 patients (75%) at a mean of 21 days (range, 1–267 days) after transplant, with CMV reactivation occurring in 6 patients, BK virus hemorrhagic cystitis in 7 patients, blood-stream positivity for bacteria in 4 patients, and SARS-CoV-2 with pneumonia and *Clostridium difficile* diarrhea in one patient each.

Thirteen patients (65%) presented with LDH levels exceeding 2 times the ULN, 10 (50%) with rUPCR ≥ 1 mg/mg, and 7 (35%) had organ dysfunction. Eighteen (90%) patients had at least two criteria for high-risk TA-TMA.

None of the adult patients discontinued CNI at the diagnosis of TA-TMA, to avoid the risk of exacerbating the concomitant GvHD. Five pediatric patients tapered or discontinued CNIs at TA-TMA diagnosis.

### Narsoplimab treatment and response

Narsoplimab was started at a mean of 3.5 days (range, 0–52) from TA-TMA onset. Patients received a median of 11 (range, 4–34) doses. Three patients responded after 8 doses. Thirteen patients received >8 doses, with 10 patients achieving a complete response after a median of 6 additional doses (range, 2–26). In 2 non-responders, the treatment was stopped after 4 and 5 doses, respectively, due to death. All infusions were well tolerated, and no signals of concern were reported.

Overall, the response rate was 65% (Table 3). Response rate was 62% (8/13) in adults and 71% (5/7) in pediatric patients. Improvement in laboratory TMA markers occurred in all responders. Before and after narsoplimab, median hemoglobin values were 9.1 g/dL (7.7–12.3) and 9.9 g/dL (8–12.4); median platelet count was 16,000 per microliter (range, 13,000–62,000) and 44,000 per microliter (range, 18,000–413,000), median LDH values were 531 U/L (range, 237–1201) and 324 U/L (range, 184–1061), respectively. The median time to response was 53 days (range, 9–133).

**Table 3:** Response to narsoplimab treatment.

Variable	Overall (N = 20)	Adults (N = 13)	Pediatrics (N = 7)
Responders, No. (%)	13/20 (65)	8/13 (62)	5/7 (71)
*Improvement in TMA markers*			
Platelet count > 30,000 per microliter or 3× baseline and no platelet transfusion, No. (%)	13/20 (65)	8/13 (62)	5/7 (71)
LDH < 1.5 × ULN, No. (%)	13/20 (65)	8/13 (62)	5/7 (71)
*Improvement in organ function*			
Freedom from transfusion^a^			
Freedom from platelet transfusion, No. (%)	13/19 (68)	8/13 (62)	4/6 (67)
Freedom from RBC transfusion, No. (%)	13/19 (68)	8/13 (62)	4/6 (67)
Improvement of renal function^b^			
rUPCR < 0.2 mg/mg, No. (%)	5/16 (31)	1/10 (10)	4/6 (67)
Reduction of creatinine >40% or creatinine below the upper limit of normal and reduction of creatinine >20% or discontinuation of renal replacement therapy, No. (%)	9/20 (45)	6/13 (46)	3/7 (43)
Improvement of gastrointestinal function^c^, No. (%)	8/11 (73)	6/8 (75)	2/3 (67)
Improvement of neurologic function^d^, No. (%)	2/6 (33)	2/4 (50)	0/2 (0)

Values show absolute numbers of patients who responded to narsoplimab treatment, with the corresponding percentage relative to the total population (20 patients, 13 adults and 7 pediatrics).

*RBC* red blood cells, *LDH* lactate dehydrogenase, *rUPCR* random urine protein-to-creatinine ratio, *ULN* upper limit of normal.

^a^One pediatric patient, who responded to treatment, was not transfusion dependent at TA-TMA diagnosis.

^b^One pediatric patient, who did not respond to treatment, had normal renal function at TA-TMA diagnosis. rUPCR at the end of treatment was not available for 3 patients.

^c^At TA-TMA diagnosis, 11 patients had gastrointestinal involvement. Improvement of gastrointestinal function was assessed using GI measures in the MAGIC criteria.

^d^At TA-TMA diagnosis, 6 patients had neurological symptoms. Improvement of neurologic function was defined as resolution of neurologic symptoms such as stroke, posterior reversible encephalopathy syndrome, seizures, confusion, or weakness.

Nineteen (95%) patients were transfusion dependent at diagnosis. All responding patients were free from platelet and red-blood-cell transfusions at the end of the treatment.

Following Khaled’s criteria of renal function response [24], 9 out of 20 patients responded (45%), while the rUPCR value improvement was more impressive in pediatric than adult patients.

Among the 13 patients who responded to narsoplimab, 8 had acute GvHD with intestinal involvement of overall grade ≥ 2.

One pediatric patient had a concomitant diagnosis of poor graft function and received a second infusion of hematopoietic stem cells after 19 doses of narsoplimab. Before the stem cell boost, this patient had gained an improved renal function but no response in terms of anemia, thrombocytopenia, and LDH. The stem cell boost induced a recovery from anemia and thrombocytopenia, but a fully normalized LDH value was documented only after 30 doses of narsoplimab.

A total of 7 patients (35%) did not respond to the treatment. Within the pediatric cohort, one patient faced fatal leukoencephalopathy with persistent active TMA, leading to the discontinuation of narsoplimab after 12 administrations. Another patient died on day +36 after transplant following 5 doses of narsoplimab administered for disease progression in the context of TA-TMA, venous occlusive disease (VOD), and multi-organ failure, unresponsive to treatment with defibrotide. Two other pediatric patients initially treated with defibrotide at TA-TMA diagnosis did not respond to it. However, they subsequently started narsoplimab and responded after 8 and 12 doses, respectively. In the adult patients, nobody received other treatment for TA-TMA before narsoplimab.

Among the adults, five patients failed to respond to narsoplimab. All of them, despite intensive care, died of progressive multiorgan failure, characterized by complications such as acute respiratory failure, acute renal failure, and hemodynamic instability. At the time of death, laboratory signs of TA-TMA could still be detected. In the fifth non-responder, narsoplimab was discontinued due to inefficacy and a concomitant refractory poor graft function.

### Survival

**Fig. 2:**
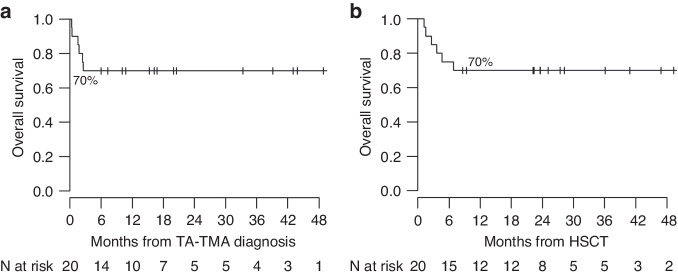
Overall survival of the cohort of pediatric and adult patients treated with narsoplimab. Overall Survival from TA-TMA diagnosis (**a**) and from transplant date (**b**).

After a median follow-up time of 13 months (range, 0.36–48.16) after TA-TMA diagnosis, the OS was 70%. One-hundred-day survival was 70% in the study population and 100% among responders (Fig. 2). None of the patients included in this study experienced TA-TMA reactivation.

## Discussion

In this retrospective study, we evaluated the safety and efficacy of narsoplimab administered under a compassionate use program in a cohort of pediatric and adult patients with high-risk TA-TMA. Our results showed that narsoplimab treatment was safe and effective.

Narsoplimab has been recently investigated in a phase 2 study for the treatment of different patterns of microangiopathy and was converted into a pivotal trial for TA-TMA only upon request to the U.S. Food and Drug Administration (FDA). The pivotal study showed that narsoplimab was safe and effective in the resolution of clinical outcomes and laboratory markers of TA-TMA with a benefit on survival [24].

While more than 130 adult and pediatric TA-TMA patients have been treated to date under the narsoplimab extended access program (EAP), our study is limited to the 20 EAP patients treated at our institutions. Therefore, it does not enable a comparison with other complement inhibitors (such as eculizumab [30–32]), occasionally used for TA-TMA treatment. Additionally, none of them are approved for the treatment of TA-TMA.

Our results are similar to those of Khaled et al., which reported a response rate of 61%. One-hundred-day survival after narsoplimab treatment was of 70% and the median OS was not reached. All the responders achieved 100-day survival (100%) and, after a median follow-up of 13 months, are alive and in remission.

Throughout treatment with narsoplimab no safety signals of concern were observed. This safety profile is particularly significant for HSCT recipients, a population prone to severe infections, especially while undergoing immunosuppressive therapy such as CNIs. At the time of TA-TMA diagnosis, physicians in charge for the treatment of pediatric and adult patients behaved differently when GvHD was concomitantly present. However, at least in adult patients, we suggest avoiding CNI withdrawal when TA-TMA is diagnosed to prevent exacerbating GvHD, which may further increase endothelial injury [33]. Notably, a retrospective cohort study conducted by the Kyoto Stem Cell Transplantation Group (KSCTG) revealed that in patients diagnosed with TA-TMA, continuing or cautiously reducing CNIs resulted in significantly improved response rates and OS compared to the strategy of transitioning from CNIs to corticosteroids (response rate, 64.7% vs. 20.0%) [34].

The diagnostic and prognostic criteria for TA-TMA remains an open issue. The diagnosis of TA-TMA is often underestimated. To enhance early recognition and diagnosis, we recommend to screen patients during the first 100 days post HSCT and to keep a high suspicion for TA-TMA even afterward, since endothelial toxicities related to infections, drugs, as well as late onset or chronic GvHD may manifest later.

The diagnostic criteria for TA-TMA are still nonspecific, and biomarkers may be needed to enable or confirm an early diagnosis of TA-TMA [7]. In a prospective study of children by Jodele et al., sC5b-9 levels exceeding the upper limit of normal range at the time of diagnosis of TA-TMA were associated with an increased risk of Non-Relapse Mortality (NRM) [35]. In adults, the sC5b-9 cutoff for prognosis is unknown, although one study suggested a cutoff of 300 mg/dL [36]. For this reason, we are now collecting serial serum and plasma samples for each patient undergoing narsoplimab treatment in anticipation of the introduction of the sC5b-9 screening test in our centers.

Finally, the histological examination remains the gold standard for TA-TMA diagnosis and ideally it should be performed whenever possible, even though the compromised clinical conditions of many patients limit the feasibility of such a diagnostic approach [37].

Our study had several limitations. These include the retrospective design, the limited number of patients treated, and their highly heterogeneous clinical presentation including the presence of both pediatric and adult cases. However, given the difficulties or the impossibility in promoting prospective control trials, real-world data are of paramount importance to understand the clinical potential of innovative treatment proposals in such a rare and often fatal disease. An additional limit is the lack of sC5-b9 data for the diagnosis of TA-TMA. It should be noted, however, that sC5b-9 is a marker of terminal complement activation and not specifically of lectin pathway activation. Future research efforts should focus on investigating whether sC5-b9 has potential utility in monitoring treatment response for a complement inhibitor that solely blocks activation of the lectin pathway.

In conclusion, our study provides real-world data on the use of narsoplimab in pediatric and adult patients with high-risk TA-TMA. Narsoplimab confirmed its effectiveness and safety across age groups, with no increased risk of infectious complications or other safety signals of concern noted. Our encouraging findings support further investigation and consideration of narsoplimab as a potential treatment for TA-TMA. The evolving landscape of TA-TMA management requires collaborative efforts to use standardized diagnostic criteria, prognostic markers, and therapeutic approaches for this severe post-HSCT complication.

## Data Availability

The datasets generated during and/or analyzed during the current study are available from the corresponding author on reasonable request.
